# Chicken Feather Waste Valorization Into Nutritive Protein Hydrolysate: Role of Novel Thermostable Keratinase From *Bacillus pacificus* RSA27

**DOI:** 10.3389/fmicb.2022.882902

**Published:** 2022-04-25

**Authors:** Chhavi Sharma, Svetlana Timorshina, Alexander Osmolovskiy, Jyoti Misri, Rajni Singh

**Affiliations:** ^1^Amity Institute of Microbial Technology, Amity University, Noida, India; ^2^Department of Microbiology, Faculty of Biology, Lomonosov Moscow State University, Moscow, Russia; ^3^Division of Animal Science, Indian Council of Agricultural Research, New Delhi, India

**Keywords:** keratinolytic protease, purification, characterization, structural modeling, scale-up, amino acid production

## Abstract

Microbial keratinases exhibit a momentous role in converting keratin biowastes into exceedingly valuable protein supplements. This study reports a novel, highly stable keratinase from *Bacillus pacificus* RSA27 for the production of pure peptides rich in essential amino acids from chicken feathers. Purified keratinase showed a specific activity of 38.73 U/mg, 2.58-fold purification, and molecular weight of 36 kDa. Kinetic studies using a chicken feather as substrate report *K*_m_ and *V*_max_ values of 5.69 mg/ml and 142.40 μg/ml/min, respectively, suggesting significant enzyme-substrate affinity/biocatalysis. Identification and *in silico* structural-functional analysis of keratinase discovered the presence of distinct amino acid residues and their positions. Besides, keratinase possesses a high-affinity calcium-binding site (Asp^128^, Leu^162^, Asn^164^, Ile^166^, and Val^168^) and a catalytic triad of Asp^119^, His^151^, and Ser^308^, known attributes of serine protease (subtilisin family). Furthermore, a scale-up to 5 L fermenter revealed complete feather hydrolysis (94.5%) within 24 h with high activity (789 U/ml) and total amino acid of 153.97 μmol/ml. Finally, cytotoxicity evaluation of protein hydrolysate resulted in negligible cytotoxic effects (1.02%) on the mammalian hepatoblastoma cell line, signifying its potential biotechnological applications.

## Introduction

Feather waste from poultry agro-industries constitutes a major environmental contaminant and accounts for several million tons of highly accumulating recalcitrant waste worldwide (Li, 2019; Mazotto et al., 2022). The disposal of such animal-derived wastes in incinerators or landfills increases both monetary and environmental costs (Wu et al., 2017). Chicken feather shows immense rigidity against physical, chemical, biological agents, and resists proteolysis by proteases like trypsin, pepsin, and papain, which is ultimately a significant obstacle in managing these costs (Qiu et al., 2022). Keratin, a rigid protein with high cysteine and disulfide bonds in β-keratin is the main component of a chicken feather that contributes to its extreme stability/resistance to degradation (McKittrick et al., 2012; Gupta and Singh, 2014; Nnolim et al., 2020a). Various high energy-consuming, non-eco-friendly thermal and chemical methods are extensively used and explored to decompose and reuse keratin (Li, 2021). But such methods ensue in the loss of some thermosensitive amino acids like tryptophan, lysine, and methionine and add-on to non-nutritive amino acids like lanthionine and lysinoalanine (Lee et al., 2016; Li, 2021).

Keratinases are extracellular-specific proteases capable of degrading keratin (Nigam, 2013; Nnolim et al., 2020a). Hydrolysis of keratinous waste (chicken feather) using microbial keratinase has gained renewed interest as it could be used in the non-polluting processes for environmental cleanup through recycling. Therefore, microbial keratinases have gained significant importance in chicken feather degradation and yield feather-feed rich in amino acids (serine, proline, and cysteine) for value addition as an animal protein supplement (Latha et al., 2016; Suman et al., 2018; Li, 2019; Qiu et al., 2022). Research suggests other promising applications of microbial keratinolytic proteases as efficient fertilizers (Singh et al., 2019), along with industrial applicability in peptide synthesis (Verma et al., 2017), therapeutic use (Gupta et al., 2017), biodegradable inexpensive thermoplastics (Jin et al., 2011), detergent, cosmetic, and leather industry (Brandelli, 2008). Thus, microbial decomposition of keratinous waste is a low-expense pivotal approach with no loss of essential amino acids and energy (Gupta and Ramnani, 2006; Brandelli, 2008).

Diverse groups of microorganisms are reported to produce keratinases (Kalia and Purohit, 2008), such as numerous *Bacillus* sp. (Gupta and Singh, 2013; Gupta et al., 2015; Dong et al., 2017; Hamiche et al., 2019; Nnolim et al., 2020b; Almahasheer et al., 2022; Devi et al., 2022; Mazotto et al., 2022), *Aspergillus oryzae* (Farag and Hassan, 2004), *Paecilomyces marquandii* and *Doratomyces microsporum* (Gradisar et al., 2005), *Streptomyces* sp. (Tatineni et al., 2008), *Stenotrophomonas maltophilia* (Cao et al., 2009), *Myrothecium verrucaria* (Moreira-Gasparin et al., 2009), *Thermoactinomyces* sp. RM4 (Verma et al., 2016), and *Fusarium* sp. (Calin et al., 2017). The limitations of existing keratinolytic enzymes constitute their low efficiency on complex feather substrates and low stability (Vidmar and Vodovnik, 2018) and suggest an intensive hunt for novel safe substitutes. Also, expanding the enzyme production process from a laboratory-scale unit to a commercial is challenging as it shows intricacy in evaluating factors such as media components, substrates, and physiological conditions during cultivation. Till date, few scale-up studies have been reported to accomplish almost complete chicken feather degradation (Peng et al., 2019).

Therefore, our research intends to suggest a novel keratinase by *Bacillus pacificus* RSA27. The enzyme showed distinct activity with high stability within a wide pH and temperature range along with metal ions/inhibitors/surfactants. Our study further reports the successful purification, characterization, and *in silico* structural-functional analysis of keratinase. As per our knowledge, this is one of the few studies which present keratinase as a potential alternative for biotransformation of chicken feathers into hydrolysate enriched with high-protein content. The scale-up of enzyme production was successfully studied in 5 L fermenter with complete chicken feather degradation within 24 h of cultivation. Henceforth, the research reveals a highly potent/stable keratinase ensuing high-protein content and might serve as an effectual alternative for chicken feather waste management.

## Materials and Methods

### Ethical Clearance

Chicken feathers were collected from a small unit of poultry production (Noida, Uttar Pradesh, India) and no live animal or human subjects were sacrificed. We have not used any live material, which could raise any ethical issue, and therefore, the study did not require an ethics review process.

### Chemicals and Reagents

All the chemicals and reagents used in the study were purchased from Sigma Aldrich, St. Louis, MO, USA.

### Inoculum Source, Culture Conditions, and Production of Keratinase

Keratinase-producing strains were isolated from soil samples (12–15 cm deep) collected from a poultry-feather dumping site at Ghazipur (New Delhi, India). Soil samples collected in sterile screw-capped vials were serially diluted and 100 μl of each dilution was spread on a sterilized nutrient agar medium. The plates were kept at 37°C for 24 h. The colony obtained on plates was screened for keratinase production by cultivating it in a sterile media containing peptone (1%), glucose (1%), KH_2_PO_4_ (0.9%), and K_2_HPO_4_ (0.3%) enriched with a chicken feather (0.5%) as substrate and microbial isolates (1%). Feathers were rinsed with tap water followed by deionized water to remove dust particles and unwanted skin, which were further air-dried at normal room temperature before their addition to the culture medium and autoclaving. The keratinolytic potential of strains was evaluated by incubating the medium containing cultures at temperature 37°C and 180 rpm for 20 days. The strains were finally screened on the basis of their feather degrading efficiency with respect to time and the highly potent strain was used for further study.

### Degradation Rate and Keratinolytic Activity of the Potent Screened Strain

The rate of degradation and keratinolytic activity of the selected isolate was further examined. Unhydrolyzed chicken feathers were harvested at fixed time intervals of 12, 24, 36, and 48 h of incubation and filtered through Whatman filter paper 1. These were then thoroughly washed with deionized water to remove culture cells and other soluble materials. The feathers were further oven-dried overnight at 60°C and the degradation rate of the screened strain was determined [chicken feather degradation (g/L) = dry weight of feathers before degradation—dry weight of feathers after degradation] using analytical laboratory weighing balance. Keratinolytic activity was determined using a chicken feather as substrate. The cell-free extract (after centrifugation at 10,000 rpm for 10 min) of keratinase production medium was used for the assay. The reaction mixture comprised keratinase (100 μl) and glycine-NaOH buffer (4.9 ml, 0.5 M, pH 9) with chicken feather (0.5%) as substrate was incubated for 1 h at 60°C. Trichloroacetic acid (4 ml of 5%) was employed to stop the reaction. The reaction mixture was filtered and the filtrate was spectrophotometrically measured at 280 nm. One unit (U/ml) of keratinase production was defined as an increase in 0.01/h absorbance at 280 nm under the assay conditions as described above.

### Scanning Electron Microscopy

Feather hydrolysis achieved by selected inoculum was pictured through SEM. The digested feathers were collected at fixed time intervals (0, 24, and 48 h) and washed thoroughly with sterile distilled water. The samples were further dried and coated with a thin gold-palladium layer using a gold sputter coater. SEM analysis was executed using Leo 435VP Microscope at an accelerating voltage of 7–21 kV (All India Institute of Medical Sciences, New Delhi, India).

### Identification of Microbial Strain

The potent selected isolate inducing chicken feather degradation was then identified for its morphological, biochemical, and molecular characteristics through Bergey's brochure for identification of bacteriology and 16S rDNA sequencing. Genomic DNA extraction of the strain was performed using a modified phenol-chloroform extraction technique. The ~1.5 kb, 16S-rDNA fragment was amplified using high-fidelity PCR polymerase in DNA Thermal Cycler (Mastercycler pro, Eppendorf). PCR amplification reaction mixture (100 μl) comprised 1 μl of template DNA, 400 ng of 16S forward primer (degenerated 5′ AGAGTTTGATCMTGGCTCAG 3′), 400 ng of 16S reverse primer (degenerated 5′ TACGGYTACCTTGTTACGACTT 3′), 4 μl of dNTPs (2.5 mM), 10 μl of Taq DNA Polymerase Assay Buffer (10×), 1 μl of Taq DNA Polymerase Enzyme (3 U/μl), and water. PCR amplification, agarose gel electrophoresis, purification of amplified DNA, and sequencing were performed as per the standardized protocol of Sharma et al. (2020). The obtained sequence was then subjected to a nucleotide blast at NCBI (https://blast.ncbi.nlm.nih.gov) and identified to species level. Evolutionary analyses were conducted through multiple sequence alignment by Clustal Omega and the maximum likelihood method. Bioinformatic tool: Molecular evolutionary genetic analysis software (MEGAX64.exe) was used to create the phylogenetic tree.

### Purification of Keratinase

The keratinase from *B. pacificus* RSA27 was purified through precipitation and chromatographic techniques to procure its purest form. The fermented medium was centrifuged at 6,000 rpm for 30 min at 4°C to remove cell debris. The protein hydrolysate (supernatant) was then treated with chilled ethanol (95%) and incubated at −20°C overnight. The mixture was centrifuged at 6,000 rpm for 15 min to procure the precipitated proteins and further purified using gel filtration chromatography. The proteins were dissolved in phosphate buffer (50 mM, pH 7.5) and loaded on Sephadex G-75 column (50 × 15 mm), which was pre-equilibrated with Tris-HCL buffer (50 mM, pH 7.5). Protein fractions were eluted at a flow rate of 1.0 ml/min with Tris-HCL buffer (50 mM, pH 7.5) and assayed for their activity and protein content (Bradford assay). Active fractions were pooled and further purified through ion-exchange chromatography using the Q Sepharose column (65 × 10 mm). The column was pre-equilibrated with glycine-NaOH buffer (10 mM, pH 9), and pooled active fractions were subjected to the column for purification. Fractions were eluted with a linear gradient of NaCl (0.6 M) at a flow rate of 1.0 ml/min. The fractions with keratinolytic activity were pooled, freeze-dried, and examined for its activity/protein concentration and molecular weight (SDS-PAGE).

### Characterization of Keratinase

#### Effect of pH and Temperature on Activity and Stability of Keratinase

The optimal pH of keratinase activity was determined using different buffers of pH values from 4 to 12. Buffers *viz*. citrate phosphate for pH 3.0–6.0, Tris-HCL for pH 7.0–8.0, and glycine-NaOH for pH 9.0–12.0 were used. The enzyme was incubated for 1 h with 0.5% chicken feather as substrate and thereafter assayed for its activity. To evaluate the optimal temperature of enzyme activity, keratinase was incubated for 1 h with 0.5% chicken feather as substrate at different temperatures ranging from 20 to 100°C and thereafter assayed for its activity. Furthermore, we have also evaluated the stability of keratinase at different pH values of 3–12 and temperatures ranging from 20 to 80°C. The enzyme was preincubated at the aforementioned pH and temperatures for 2 h without substrate and then assayed for its activity after every 30 min of time interval (30–120 min).

#### Keratinase Activity in the Presence of Metal Ions, Inhibitors, and Surfactants

The keratinolytic activity of the enzyme in the presence of potential metal ions, inhibitors, and surfactants was evaluated. Metal ions such as Na^+^, K^+^, Zn^2+^, Co^2+^, Ca^2+^, Fe^2+^, Mn^2+^, Cu^2+^, Mg^2+^, and Hg^2+^ and inhibitors such as ethylene-diamine tetra acetic acid (EDTA), β-mercaptoethanol, phenylmethylsulfonyl fluoride (PMSF), urea, indole-3-acetic acid (IAA), N-bromosuccinimide, 1,10-phenanthroline, and dithiothreitol (DTT) of 2 and 5 mM concentration were used. Besides, the effects of various (1 %) surfactants *viz*. saponin, sodium cholate, sodium dodecyl sulfate (SDS), Tween 80, and Triton X 100 on enzyme activity were also studied. The keratinase was preincubated for 1 h with the aforementioned chemical reagents and then assayed for its activity at optimal pH of 9 and temperature of 60°C. The sample without any chemical reagent was treated as a control.

### Enzyme Kinetics

GraphPad Prism 9.0 Ink was used to evaluate kinetic parameters (*K*_m_ and *V*_max_) of keratinase using non-linear regression, Michaelis-Menten curve with various concentrations of a chicken feather as substrate (0.5–5.0 mg/ml). The experiments were conducted under optimum temperature and pH conditions.

### *Ker* Gene Sequencing and Keratinase Prediction

The identification of gene (*ker*) responsible for the production of keratinase was identified through sequencing. Forward primer 5′-AAAAGGAGAGGGTAAAGAGT-3′ and reverse primer 5′-AGCAGGTATGGAGGAGCCTG-3′ were used for PCR amplification. Amplification reaction, gel electrophoresis, and sequencing were performed as per the standardized protocol of Gupta et al. (2017). Bioinformatics tool ExPASY (http://web.expasy.org/translate) was used to translate the obtained *ker* gene sequence into the amino acid sequence to further get an insight into the potent keratinase responsible for feather degradation. The deduced sequence was analyzed using PSI-BLAST (https://blast.ncbi.nlm.nih.gov/Blast.cgi?PAGE=Proteins), aligned using CLUSTAL W (http://www.ebi.ac.uk/clustalw) for the identification of conserved residues and sequence logos were generated using WebLogo 3.7.4 program (http://weblogo.threeplusone.com/) for a clear alignment of identified conserved domains.

### Structural Modeling and *in vitro* Validation

The three-dimensional (3D) structure of keratinase was predicted using homology modeling tools—Swiss-model (https://swissmodel.expasy.org/) and Phyre2 (http://www.sbg.bio.ic.ac.uk/phyre2). Modeling server Swiss-model predicted one calcium-binding site (Ca1) of keratinase, which was visualized using Ligplot+ version 2.2 (https://www.ebi.ac.uk/thornton-srv/software/LigPlus/). *In vitro* validation evaluating the effect of Ca ions on the thermostability of keratinase was further studied by incubating keratinase for 2 h with 5 mM of CaCl_2_ at different temperatures (20–80°C). Keratinolytic activity was performed as per our standard protocol. A reaction mixture without CaCl_2_ served as a control.

### Scale-Up in a Laboratory 5-L Batch Fermenter

The *in vitro* feather degradation was scaled up in a 5 L continuous stirred tank bioreactor (Biostat-B-Lite, Laboratory fermenter system, Sartorius, Germany). A total of 3 L of optimized media was sterilized and inoculated with 2% of overnight grown culture and 0.5% chicken feather waste. The fermentation was carried out for 24 h at 37°C at 180 rpm agitation, uncontrolled pH, and sterile air with a constant rate of 2 vvm. Samples were withdrawn at fixed intervals (2 h) and analyzed for keratinase production and change in pH with respect to time. The fermentation parameters such as temperature, pH, and airflow were continuously monitored throughout the process.

### Amino Acid Profiling

The amino acid profiling of *B. pacificus* RSA27 keratinase was accomplished using cell-free extract of digested feathers in triplicates at the time interval of 0, 12, and 24 h using high-performance chromatography (Agilent 1100 HPLC, Santa Clara, United States) and was performed as per the standardized protocol of Gupta and Singh (2014).

### *In vitro* Cytotoxicity Evaluation of Protein Hydrolysate

Cell-free supernatant 0.09–50% (v/v) of digested feathers was analyzed for cytotoxicity using mammalian hepatoblastoma cell line HepG2 (8 × 103 cells/well). Doxorubicin hydrochloride (0.10–5.00 μM) and untreated cells served as positive and negative controls, respectively. The cells were then incubated at 37°C for 72 h, and cytotoxicity was assessed using MTT [3-(4,5-dimethylthiazol-2-yl)-2,5-diphenyltetrazolium bromide] assay. MTT (20 μl of 5 mg/ml) was added to each well and incubated for 3 h at 37°C. The supernatant was aspirated and dimethyl sulfoxide (150 μl) was added to each well to dissolve formazan crystals. Microplate reader (BioTek Winooski, VT, USA) was used to measure absorbance at 540 nm, and cytotoxicity (%) was determined.

## Results and Discussion

### Chicken Feather Degradation Rate of Keratinase

**Figure 1:**
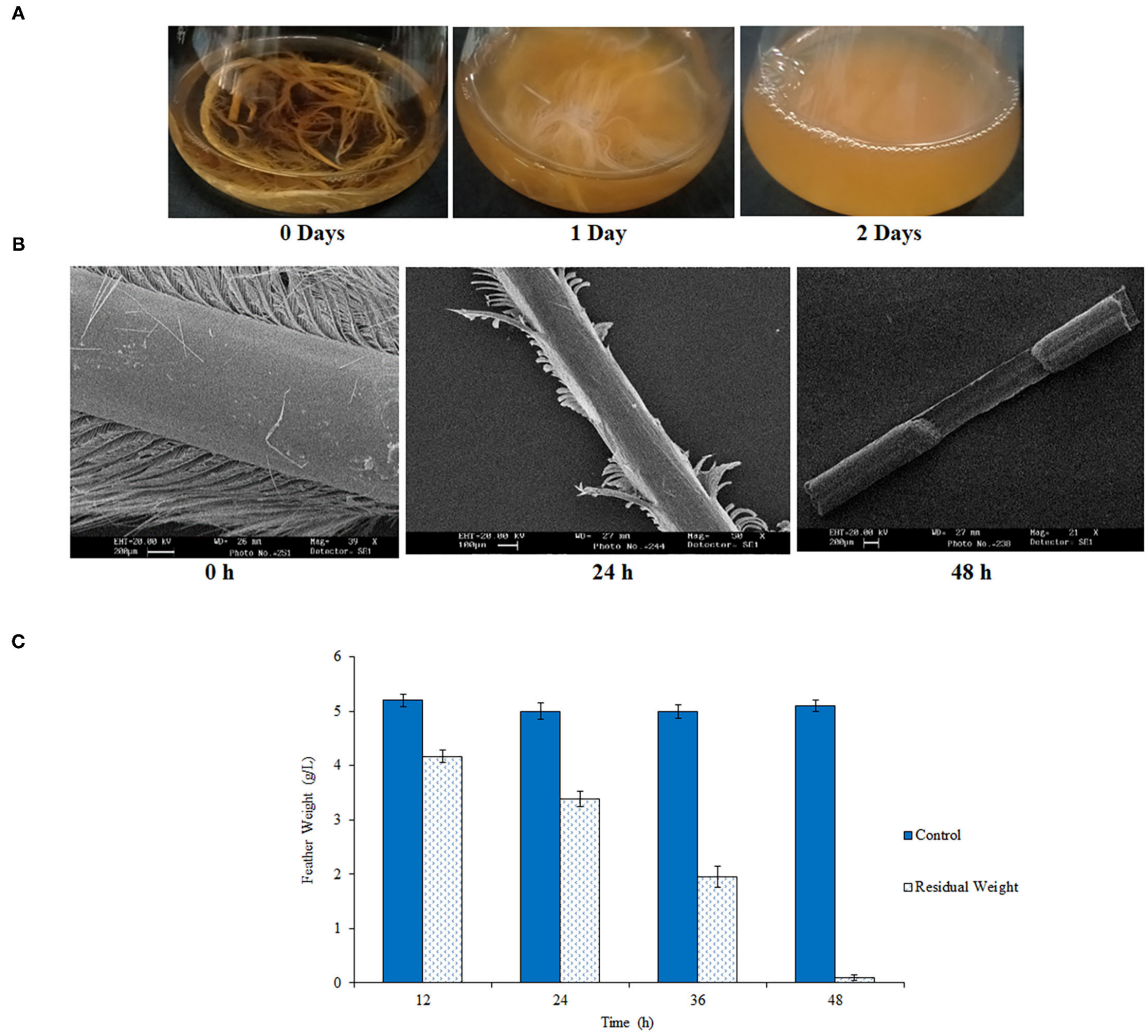
Chicken feather degradation by RSA27. **(A)** RSA27 efficacy with respect to time at 180 rpm and 37°C. 0 days: control, 1 day: RSA27 initiated feather degradation after 24 h, 2 days: complete degradation of feathers after 48 h. **(B)** SEM of feather degradation at 0 h (intact feather: control), 24 h (degradation of feather barbs), and 48 h (calamus degradation leading to complete degradation of the feather). **(C)** A residual dry weight of feathers after 12, 24, 36, and 48 h.

In total, 44 strains out of the 200 isolates exhibited keratinolytic ability. Out of which feather degradation with 24 strains was observed in 15–20 days, 19 strains in 5–10 days, and 1 strain in 2 days. The highest feather degrading capacity was observed with isolate RSA27, which initiated the fastest feather degradation within 24 h and completely degraded feather within 48 h of incubation at 180 rpm and temperature 37°C (Figure 1A). The breakdown of a chicken feather was visualized using SEM after 0, 24, and 48 h of incubation. Incubation for 24 h resulted in degradation of feather barbs, whereas calamus degradation was observed after 48 h of incubation, leading to a complete breakdown of chicken feathers (Figure 1B). A similar result was reported by Gupta and Singh while estimating chicken feather hydrolyzing ability of two *Bacillus subtilis* strains E163 and E165 after 24, 48, and 72 h of cultivation (Gupta and Singh, 2014). In another study, keratinase from *B. subtilis* 8 resulted in a complete breakdown of the chicken feather-shaft structure within 48 h when analyzed and identified through SEM (He et al., 2018). A significant decrease in the weight of chicken feathers hydrolyzed by RSA27 was observed with the increase in incubation time (0–48 h). The observed residual weight (g) was 4.17, 3.39, 1.95, and 0.1 after incubation of 12, 24, 36, and 48 h, respectively. Complete feather degradation was observed after 48 h of incubation (Figure 1C). The keratinolytic activity of RSA27 strain after 48 h was found to be 125.10 U/ml.

### Identification of Keratinolytic Strain

The isolate RSA27 morphologically exhibited non-translucent, white, and circular (2–3 mm in diameter) colonies. It was observed as non-motile, rod-shaped, and gram-positive *Bacillus* strain. Biochemical characterization imparted its positive catalase, gelatinase, and oxidase activities. Results were positive for citrate utilization, Voges-Proskauer test, and arginine dihydrolase while negative for lysine decarboxylase, β-galactosidase, H_2_S, indole, and acid production from arabinose, amygdalin, mannitol, sorbitol, sucrose, glucose, rhamnose, inositol, and melibiose. Such phenotypic and biochemical specifications were similar to strain *B. pacificus* EB422T of *Bacillus cereus* group (Liu et al., 2017). So, the strain was further confirmed and identified as *B. pacificus* RSA27 (NCBI Accession number MT180833.1) by 16S rDNA sequencing. Phylogenetic analysis by maximum likelihood methodology conveyed details about the distinct position and evolutionary association of *B. pacificus* RSA27 with other *Bacillus* strains (Supplementary Figure 1).

### Purification

**Figure 2:**
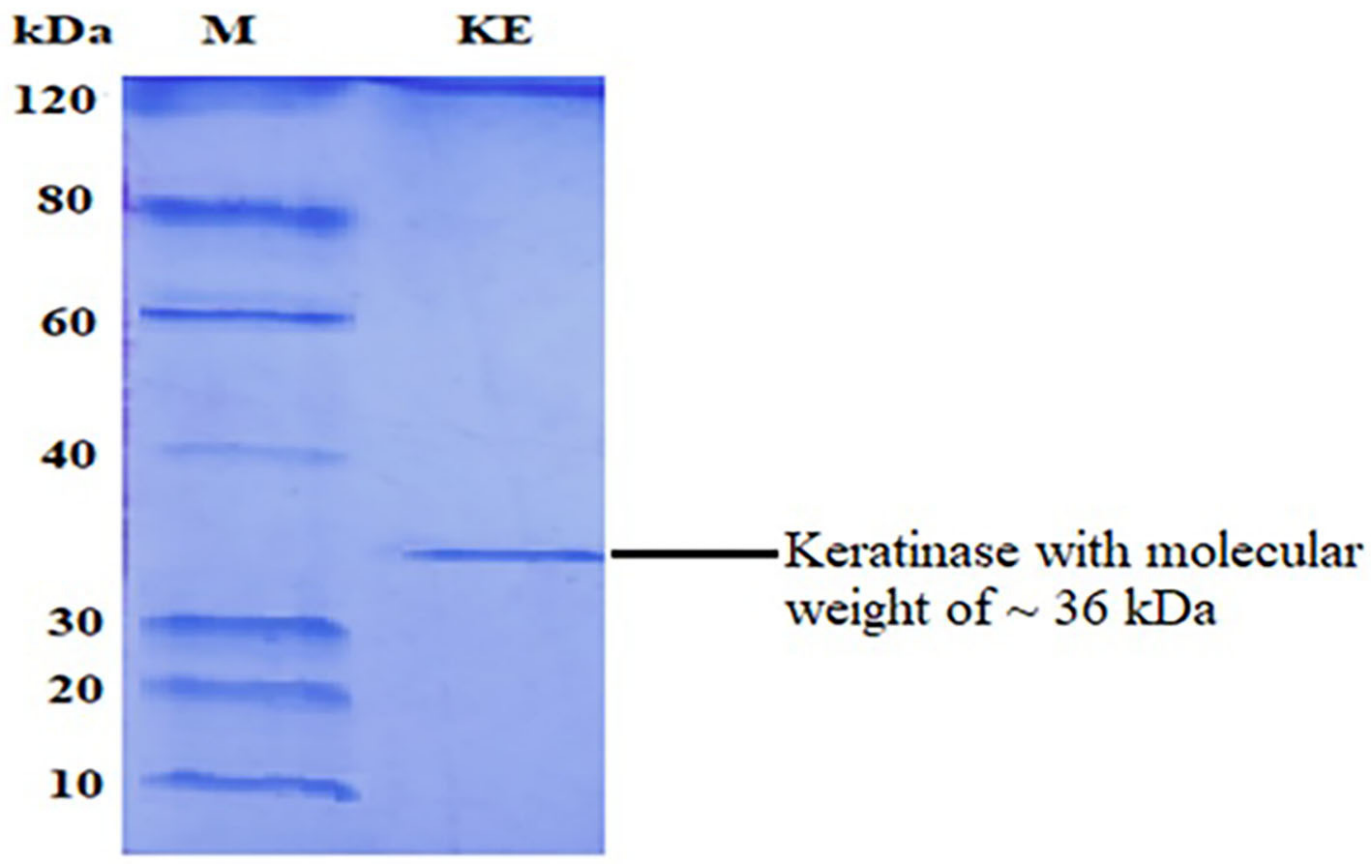
SDS-PAGE of purified keratinase. M, protein marker; KE, keratinase.

Purification of keratinase through chilled ethanol precipitation and Sephadex G-75 column resulted in 75.02% yield with 1.25-fold purification and 54.48% yield with 2.05-fold purification, respectively. Furthermore, the enzyme was 2.58-fold purified with a yield of 54.48% relative to crude keratinase by Q Sepharose (Table 1). A specific activity of 38.73 U/ml was assessed for the ultimate keratinase. The molecular weight of ~36 kDa was analyzed by SDS-PAGE (Figure 2). Literature reports numerous keratinolytic enzymes of apparently similar molecular weight of 35 kDa from *Bacillus licheniformis* (Suntornsuk et al., 2005), 42 kDa from *Microbacterium* sp. (Thys and Brandelli, 2006), and 44 kDa from *Streptomyces* sp. (Tatineni et al., 2008).

**Table 1:** Purification parameters of keratinase from *Bacillus pacificus* RSA27.

**Purification steps**	**Total activity (U/mL)**	**Total protein (mg/mL)**	**Specific activity (U/mg)**	**Yield (%)**	**Fold purification**
Crude enzyme	125.10 ± 5.21	8.34 ± 0.52	15.00 ± 1.09	100	1
Ethanol precipitation	93.85 ± 3.76	4.99 ± 0.31	18.81 ± 1.21	75.02	1.25
Sephadex G-75	68.15 ± 3.11	2.21 ± 0.17	30.84 ± 1.17	54.48	2.05
Q Sepharose	64.29 ± 2.86	1.66 ± 0.09	38.73 ± 1.24	51.39	2.58

### Characterization of Keratinase

#### Activity and Stability of Keratinase Under Varied pH and Temperature

**Figure 3:**
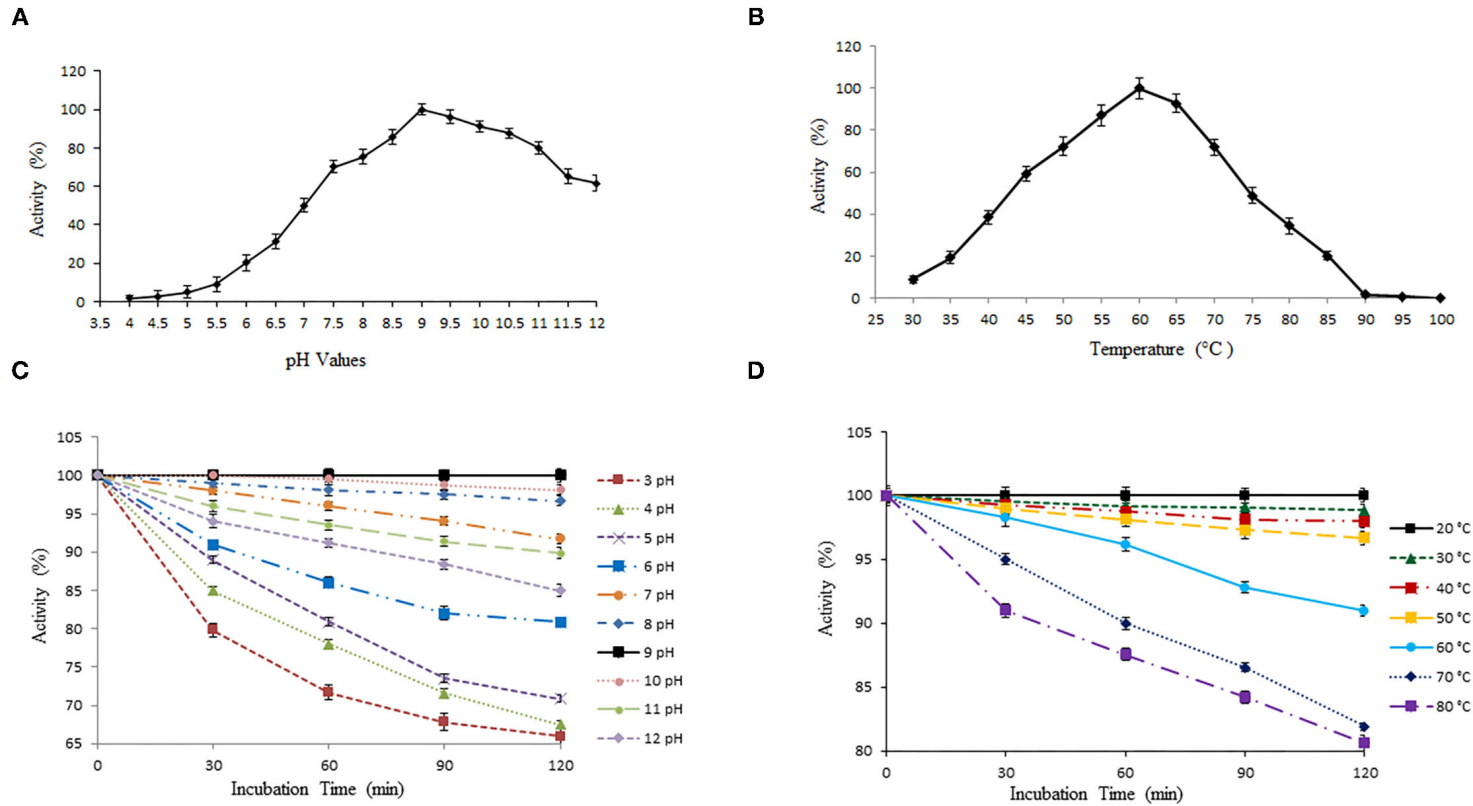
Effect of temperature and pH on activity and stability of keratinase. **(A,B)** The optimal activity of keratinase was observed at pH 9 and 60°C. **(C,D)** Keratinase exhibits stability within wide pH (3–12) and temperature (20–80°C) range.

We have further quantified the keratinase activity and stability to evaluate enzymatic hydrolysis within wide pH and temperature range. Keratinase exhibited 100% enzymatic activity with a chicken feather as substrate (0.5%) at pH 9 (Figure 3A). The keratinase retained its full stability (100%) at pH 9 even after 2 h of incubation, while a slight reduction in activity was observed from pH 3 to 8 and pH 10 to 12 (Figure 3C). Most of the reported proteases are alkaline in nature and thus beneficial for various biotechnological applications (Rahman et al., 2005; Verma et al., 2016; Pandey et al., 2019; Sharma et al., 2020). Our results are in resemblance with some other previous studies as well, which again suggests that alkaline pH alters cysteine residues to lanthionine and aids in keratinolytic activity with high industrial applicability (Rissen and Antranikian, 2001; Selvam et al., 2013). Also, *B. pacificus* RSA27 originated keratinase is stable even under acidic conditions (pH 3–6) with no major loss in activity, suggesting its utilization as an animal feed supplement, which can retain its functionality even after passing through the mammalian digestive system (Wu et al., 2017). Research supports the vision of the use of biowastes as feed, which makes the process economical (Patel et al., 2021).

The temperature profile of keratinase demonstrates optimum activity at 60°C (100%) with wide stability within 20–80°C (Figure 3B). The enzyme showed 100% stability at 20°C and a slight reduction in activity of 98.84, 97.97, 96.69, 90.98, 81.92, and 80.59% with an increase in temperature from 30 to 80°C, correspondingly (Figure 3D). Our results were in contrast with some earlier reports where after 2 h of incubation, keratinase of *Thermoactinomycetes* sp. showed 100% stability at 70°C (Verma et al., 2016). Another keratinase rMtaker was also found stable at 70°C after 2 h of incubation (Wu et al., 2017). Also, as mentioned, the maximum activity of our keratinase was observed at 60°C (with substrate) and full stability at 20°C (without substrate). Hence, it becomes necessary to mention here that the presence of protein substrates (here chicken feather) tend to increase the thermostability and activity of heated enzymes. A similar phenomenon was reported and discussed in other studies as well (Chang and Mahoney, 1995; Wu et al., 2017).

#### Keratinase Efficacy in the Presence of Metal Ions, Inhibitors, and Surfactants

**Figure 4:**
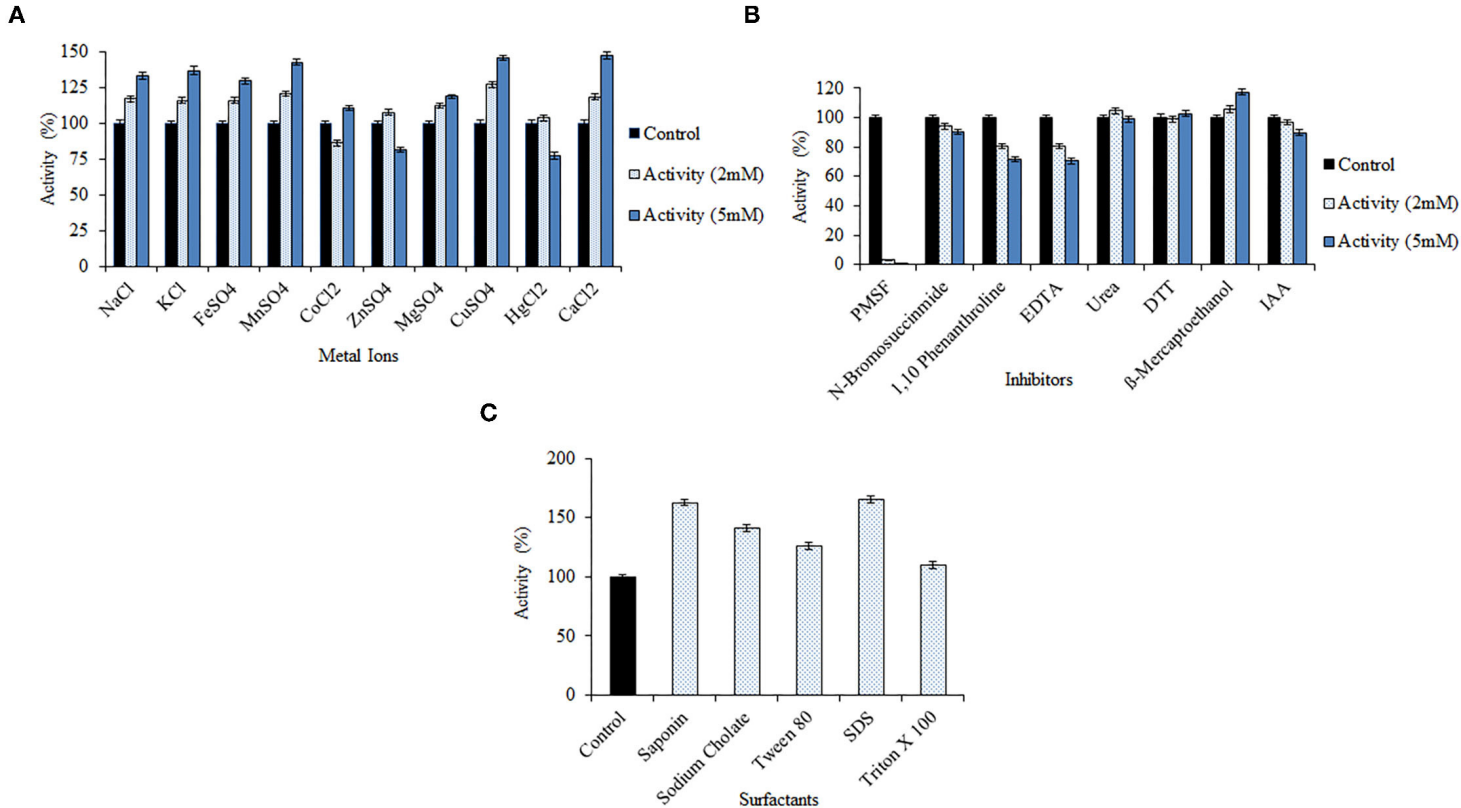
Effect of various metal ions/inhibitors/surfactants on the keratinolytic activity of the enzyme from *Bacillus pacificus* RSA27. **(A,B)** Effect of metal ions and inhibitors (2 and 5 mM). **(C)** Effect of surfactants (1% solution with water).

*Bacillus pacificus* RSA27 derived keratinase was treated with different potential metal ions/inhibitors (2 and 5 mM) and surfactants (1%), and the activity was measured using a chicken feather as substrate. Keratinase activity was significantly enhanced in the presence of monovalent *viz*. Na^+^ and K^+^ and divalent metal ions *viz*. Mn^2+^, Fe^2+^, Mg^2+^, Cu^2+^, and Ca^2+^, at both the assayed concentrations. An activity increase of 32.84, 36.85, 42.54, 29.58, 18.59, 45.99, and 47.01% was observed in the presence of 5 mM concentration of Na^+^, K^+^, Mn^2+^, Fe^2+^, Mg^2+^, Cu^2+^, and Ca^2+^, respectively. Such an outcome suggests the requirement of these metal ions for optimal activity of the enzyme (Patel et al., 2019, 2022; Kondaveeti et al., 2022). Besides, slight inhibition with 86.24% of keratinase activity was observed at low concentration (2 mM) while a slight increase of 10.32% in activity was testified at high concentration (5 mM) of metal ion Co^2+^. On the contrary, an increase of 7.51 and 3.58% in enzyme activity was observed at low concentration of Zn^2+^ and Hg^2+^, respectively. However, when used in high concentration, Zn^2+^ and Hg^2+^ resulted in the slight inhibition of keratinase activity (Figure 4A). Our results are in contrast to some previous research reports where no significant effect on keratinase activity was observed with monovalent ions (Na^+^, Li^+^, and K^+^; Jaouadi et al., 2013). However, results similar to our study were reported by Verma et al. (2016) wherein some remarkable activity of keratinase from *Thermoactinomycetes* sp. RM4 was observed in the presence of monovalent ions Na^+^ and K^+^. Another study by Yong et al. (2020) detailed the stimulation of keratinase activity with Mg^2+^ and Ca^2+^ and complete inhibition by Cu^2+^. Our study also reports high keratinase activity at both concentrations of Ca^2+^, which is normally considered a standard attribute of serine proteases (Yong et al., 2020). Furthermore, inhibition inactivity of keratinase from *Bacillus subtilis* K-5 was observed with metal ions Na^+^, K^+^, Zn^2+^, Mn^2+^, Hg^2+^, Cu^2+^, and Ca^2+^ (Singh et al., 2014).

The keratinase was found to be serine protease in nature, as the presence of both high and low concentrations of PMSF resulted in complete loss of keratinase activity. Keratinase activity of 3.07% and 0.09% was observed with 2 and 5 mM of serine protease inhibitor PMSF, respectively. Also, with the increase in the concentration of inhibitors *viz*. N-bromosuccinimide, 1,10-phenanthroline, EDTA, IAA, and urea, the activity of keratinase gradually decreased. No significant decrease in enzyme activity was observed with DTT and mercaptoethanol at their respective concentrations (Figure 4B). BsKER71 keratinase by *Bacillus subtilis* S1-4 was also strongly inhibited by PMSF and reported as serine protease (Nnolim et al., 2020b). Similar observations were stated from keratinases of *Brevibacillus brevis* US575 (Jaouadi et al., 2013) and *Aspergillus parasiticus* (Anitha and Palanivelu, 2013). Also, studies have shown that metalloprotease inhibitors (EDTA and 1,10-phenanthroline) might slightly inhibit the catalytic activity of some serine proteases, which recommends the requirement of transition metals for their hydrolysis (Nnolim et al., 2020b).

However, a significant increase in keratinolytic activity was noticed with surfactants like saponin (1.63-fold), sodium cholate (1.41-fold), Tween 80 (1.26-fold), SDS (1.65-fold), and Triton X 100 (1.09-fold) (Figure 4C). Our observations are similar to keratinase from *Bacillus polyfermenticus* B4 wherein an increase in relative activity (%) of the enzyme was observed with Triton X 100 (109 ± 5), Tween 80 (100 ± 1), and SDS (279 ± 2; Dong et al., 2017). Another surfactant stable keratinase was obtained from *Brevibacillus parabrevis* CGMCC 10798, wherein 11 and 30% increment in enzyme's activity was observed with Tween 40 and Triton X 100, respectively (Zhang et al., 2016). Remarkable keratinolytic activity was observed with saponin (1.91-fold) and sodium cholate (1.87-fold) in other scientific reports as well (Rajput et al., 2010).

### Enzyme Kinetics

**Figure 5:**
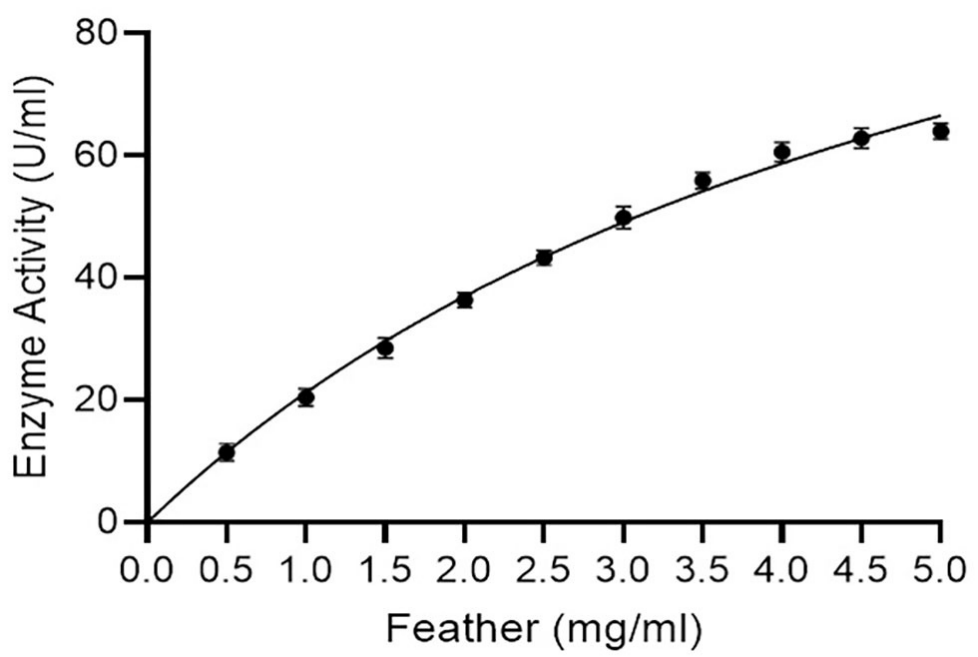
Enzyme kinetics (Michaelis-Menton plot) for keratinase using a chicken feather as substrate (*R*^2^ = 0.9902).

*K*_m_ and *V*_max_ values of keratinase using different concentrations of a chicken feather as substrate (0.5–5.0 mg/ml) were 5.69 mg/ml and 142.40 μg/ml/min, respectively, with *R*^2^ of 0.9902 (Figure 5). *K*_m_ and *V*_max_ values of keratinase SLSP-k using casein (substrate) were 0.64 mM and 420 μmol/ml/min, respectively (Gurunathan et al., 2021). Another research on alkaline protease reports *K*_m_ and *V*_max_ values of 7.0 mg/ml and 54.30 μmol/ml/min, respectively (Mushtaq et al., 2012).

### *Ker* Gene Sequencing and Prediction of Keratinase Sequence

The *ker* gene sequence study revealed the presence of 1,197 bp nucleotides encoding for 399 amino acids (Supplementary Figure 2). The longest reading frame (362 amino acids) from position 26 to 387 was chosen for further *in silico* analysis and is mentioned underneath.

MAFSNMSAQAAGKSSTEKKYIVGFKQTMSAMSSAKKKDVISEKGGKVQKQFKYVNAAAATLDEKAVKELMIPADLGKKGADRPIIKGAQSVPYGISQIKAPALHSQGYTGSNVKVAVIDSGIDSSHPDLNVRGGASFVPSETNPYQDGSSHGTHVAGTIAALNNSIGVLGVAPSASLYAVKVLDSTGSGQYSWIINGIEWAISNNMDVDASKENATFGHINSAQANDPSVSSGIVVAAAAGNEGSSGSTSTVGYPAKYPSTIAVGAVNSSNQRASFSSAGSELDVMAPGVSIQSTLPGGTYGAYNGTSMATPHVAGAAALILSKHPTWTNAQVRDRLESTATYLGSSFYYGKGLINVQAAAQ

**Figure 6:**
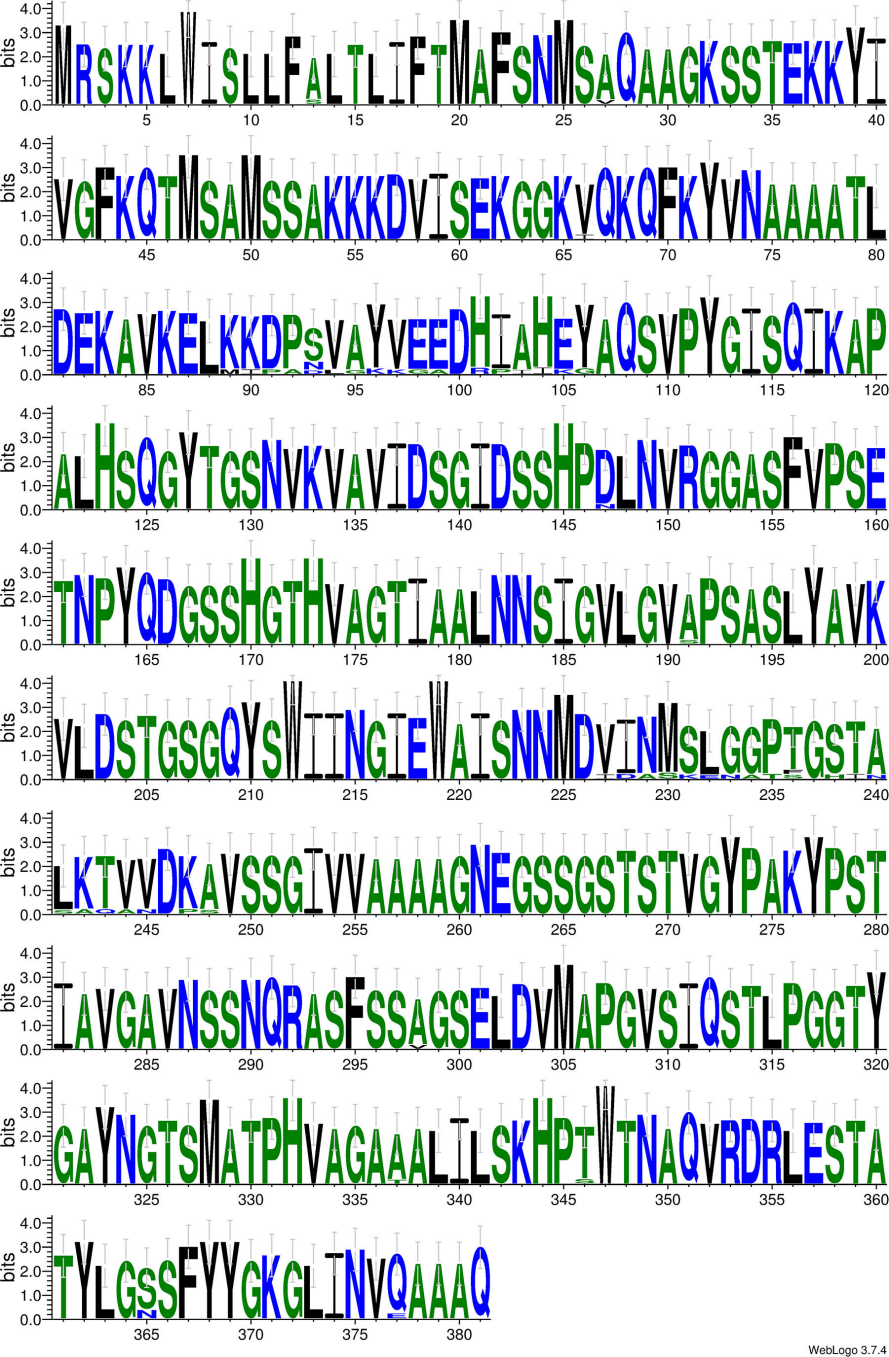
Weblogo for conserved domain analysis of keratinase. Blue, green, and black colored are hydrophilic, neutral, and hydrophobic residues, respectively. The relative frequency of each residue is symbolized by height of symbol within stack and overall height of stack demarcates degree of conservation.

Prosite search revealed that keratinase belongs to the subtilase family (serine protease) showing three distinct active sites ^115^VAVIDSGIdssH^126^ (Asp active site), ^151^HGThVAGtIAA^161^ (His active site), and ^306^GTSmAtPhVAG^316^ (Ser active site). Further, PSI-BLAST depicted that the top 10 homologous enzymes were used for conserved domain analysis of keratinase. Subtilisin AprE enzymes from *Bacillus* (WP_041335567.1 and WP_017695350.1) with a maximum score of 631, a total score of 631, query cover of 100%, *e*-value of 0, the identity of 90.06%, and length of 381 were detected with maximum sequence homology with keratinase. Additionally, moderate sequence similarity was observed with keratinase from *Bacillus subtilis* (AIY62812.1) with a maximum score of 630, a total score of 630, query cover of 100%, *e*-value of 0, the identity of 90.06%, and length of 362. A clear alignment of Clustal Omega identified that conserved domains were generated using Weblogo. Results indicate that six large stretches of highly conserved domains in homologous regions were observed from positions (Gln^28^-Lys^65^, Gln^67^-Leu^88^, Ala^107^-Pro^146^, Leu^148^-Asp^226^, Val^249^-Ser^297^, and Gly^299^-Ala^336^) and low keratinase conserved amino acids at Met^89^-Ala^99^, Arg^100^-Gly^106^, Asp^228^-Phe^236^, His^238^-Asn^245^, Pro^247^, and Ser^248^ (Figure 6; Supplementary Figure 3). Alignment of a novel keratinase KerUS from *Brevibacillus brevis* US575 with homologous enzymes revealed distinct keratinase residues in the mature region at Glu^13^, Lys^51^, Ala^65^, Ser^87^, Asp^104^, Pro^129^, Asn^130^, Phe^159^, Thr^162^, Asp^172^, Gly^175^, Ser^182^, Asn^186^, Ala^219^, Val^233^, Tyr^238^, Thr^242^, and Asn^243^ (Jaouadi et al., 2013). Another study of a novel thermostable keratinase from *Deinococcus geothermalis* reports numerous conserved residues along with four conserved stretches from Asp^163^-Ile^166^, Gly^195^-Thr^203^, Asn^252^-Gly^257^, and Gly^346^-Met^349^ (Tang et al., 2021). The outcomes thus suggest that keratinase exhibits no absolute similarity with already reported *Bacillus* sp. protein and is a novel keratinolytic enzyme with significant feather hydrolysis potential.

### Structural Modeling and *in vitro* Validation

**Figure 7:**
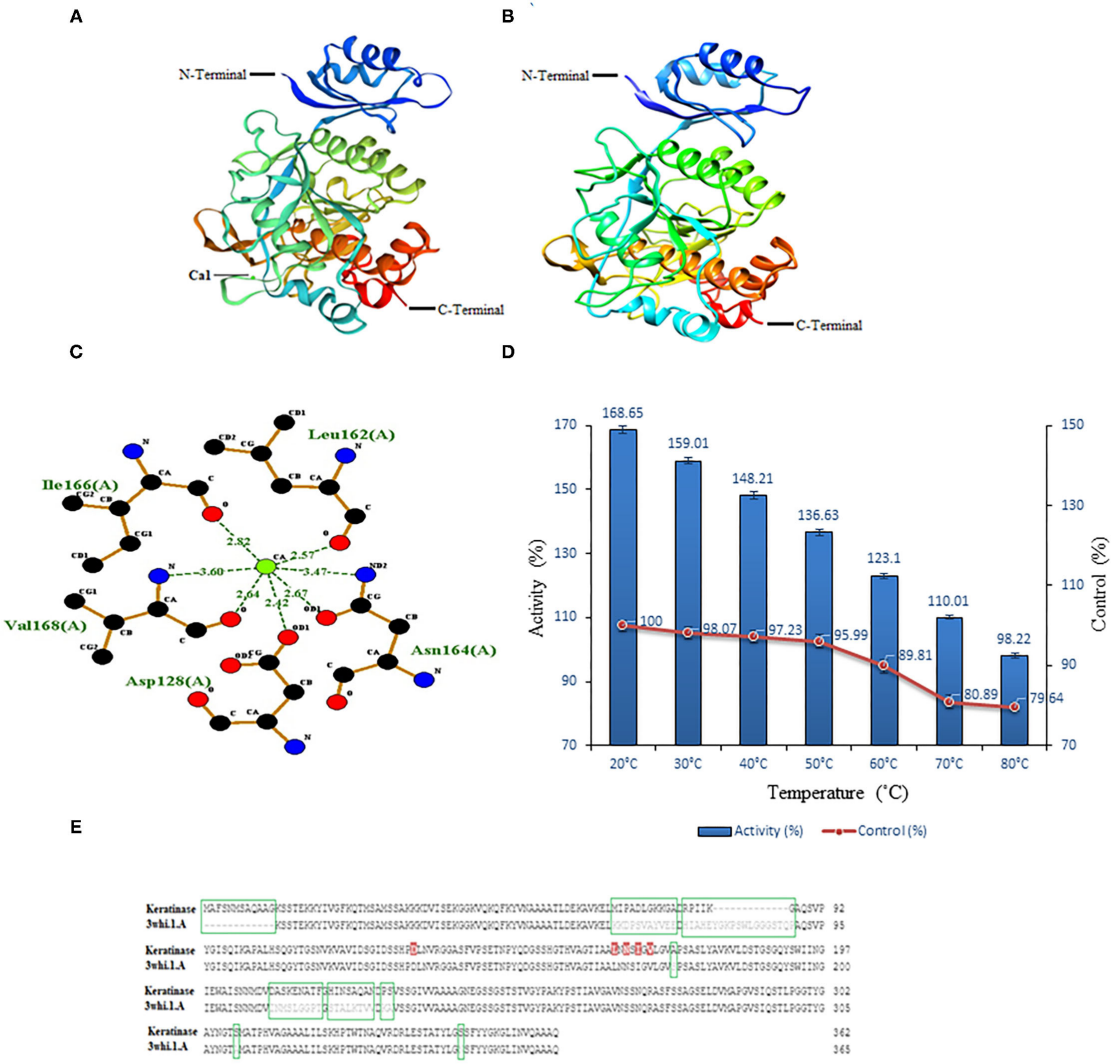
Modeled structures of keratinase and model-template alignment. **(A)** 3D structure of keratinase modeled using Swiss-model. The green ball here represents calcium ions. **(B)** 3D structure of keratinase modeled using Phyre2. **(C)** *In silico* analysis of Ca1 binding site. Two-dimensional view of interactive residues of keratinase with Ca1 (Asp^128^, Leu^162^, Asn^164^, Ile^166^, and Val^168^) along with the distance between target atom and Ca1. **(D)** *In vitro* effect of CaCl_2_ (5 mM) on keratinase activity at different temperatures (20–80°C). **(E)** Encircled (green) are missing/dissimilar residues of keratinase in comparison to template and red marked are the predicted Ca1-binding sites of keratinase.

Swiss-model modeled keratinase structure with GMQE (0.86) and QMEAN (−2.01) and predicted 88.86% identity with “3whi.1.A” (subtilisin E, Crystal structure of unautoprocessed form of IS1-inserted pro-subtilisin E; Figure 7A). Besides, another modeling server Phyre2 predicted keratinase structure with 100% confidence, 96% coverage, and 89% identity with template “c3whiA” (Figure 7B). The modeled keratinase was found to exhibit Ca1 (calcium-binding site) and amino acid residues Asp^128^, Leu^162^, Asn^164^, Ile^166^, and Val^168^ were present amid 4 Å for ligand contacts (Figure 7C). Subtilisin enzymes from *Bacillus* sp. have two high-affinity Ca1 and low-affinity Ca2-binding sites, which have (specifically Ca1) aids in protection against autolysis and thermal stability (Sharma et al., 2021a,b). Our *in silico* study supports the *in vitro* characterization results wherein an increase in activity was observed in the presence of CaCl_2_ (2/5 mM). The effect of Ca ions on thermostability was further analyzed to draw conclusive results. A significant increase of 68.65, 60.94, 50.98, 40.64, 33.29, 29.12, and 18.58% was examined in the presence of CaCl_2_ (5 mM) at temperatures 20, 30, 40, 50, 60, 70, and 80°C, respectively, thus validating our *in silico* outcome (Figure 7D). Enhanced thermostability of proteases M179 and AprE176 from *Bacillus subtilis* HK176 was accessed with Ca ions wherein M179 retained 36% of its activity after 5 h at 45°C while AprE176 retained 11% activity (Jeong et al., 2015). RFEA1 from *B. cereus* RSA1 showed 30.87% of increase in its activity in the presence of CaCl_2_ and an increased thermostability of 10.58, 21.12, 26.71, 30.17, 36.98, 41.25, and 45.65% at temperatures 80, 70, 60, 50, 40, 30, and 20°C, respectively (Sharma et al., 2021a). In another study, *Bacillus subtilis* DC27-produced enzyme was reported with enhanced activity (122.02 ± 5.71%) in the presence of 5 mM Ca ions (Hu et al., 2019).

Model-template alignment revealed that stretch of residues from Met^1^ to Gly^12^ is lacking in the template with respect to keratinase and from Tyr^77^ to Gln^89^ is lacking in keratinase with respect to the template. Besides, a large number of dissimilar residues (Met^70^-Ala^80^, Arg^82^-Lys^86^, Gly^87^, Ala^172^, Asp^209^-Phe^217^, His^219^-Asn^216^, Pro^228^, Ser^229^, Ser^308^, and Ser^346^) were reported in keratinase in comparison to a template. Additionally, huge dissimilarity in positions of residues was observed amid keratinase and its template (Figure 7E). Research suggests that despite high identity, homologous enzymes might exhibit distinct residues and lack some stretches of amino acids (Sharma et al., 2021a).

### Scale-Up and Amino Acid Profiling

Scale-up to a 5 L fermenter was performed to improve efficiency and outstretch the broad industrial conditions of chicken feather hydrolyzing novel *B. pacificus* RSA27 isolate. Predictably, all chicken feathers degraded within 24 h of inoculation with a degradation rate of 94.5% and left with the remaining 0.825 g undecomposed feather in the culture, which is extremely difficult to decompose. Keratinase production started within 2 h of culture and retained its maximum activity (789.00 U/ml), which was 6.31-fold increment than the original activity (125.01 U/ml). A gradual increase in pH (9.20) was observed with respect to time (Table 2). Besides, the scale-up cultivation in a 5 L bioreactor has led to an increase in efficiency of feather hydrolysis by lowering degradation time to 24 h, which is half the degradation time in 100-ml Erlenmeyer flasks. Therefore, such large-scale optimization using novel *B. pacificus* RSA27 exhibits significant potential for industrial poultry feather waste degradation. In recent research, 81.8% rate of chicken feather degradation was observed in a 3-L fermenter and an increase in chicken feather degrading efficiency was accomplished by reducing the degradation time to half the actual time (Peng et al., 2019). Bioreactor scale-up of enzyme production has also been performed and reported in many other previous reports, wherein Ni et al. (2011) stated five-fold increase in keratinase production in 5 L bioreactor using optimized medium. Similarly, Fang et al. (2013) has observed enhanced production of keratinase in fed-batch conditions using keratin waste (117.7%).

**Table 2:** Chicken feather degradation in 5 L fermenter depicting change in keratinase activity and pH in reaction system with due course of time.

**Time (h)**	**Keratinase activity (U/mL)**	**pH**
0	0	7.20 ± 0.05
2	21.57 ± 1.01	7.35 ± 0.08
4	47.20 ± 2.33	7.55 ± 0.10
6	100.21 ± 3.91	7.69 ± 0.10
8	189.36 ± 5.95	7.90 ± 0.15
10	248.51 ± 7.15	8.00 ± 0.17
12	356.98 ± 8.41	8.25 ± 0.18
14	441.47 ± 8.97	8.41 ± 0.20
16	509.92 ± 8.94	8.70 ± 0.25
18	627.23 ± 9.76	8.85 ± 0.22
20	789.00 ± 10.43	9.10 ± 0.35
22	780.87 ± 10.02	9.18 ± 0.39
24	768.04 ± 10.18	9.20 ± 0.41

The hydrolyzed chicken feather consists of protein β-keratin (91%), which was analyzed through high-performance liquid chromatography at the time intervals of 12 and 24 h of the degradation process. The concentration of hydrolyzed sample (12 and 24 h) is depicted in Table 3, wherein the total composition of amino acids was observed 153.97 μmol/ml after 24 h of hydrolysis. An increase in the concentration of aspartic acid (2.45-fold), tyrosine (5.08-fold), valine (6.74-fold), methionine (2.32-fold), phenylalanine (3.29-fold), isoleucine (2.80-fold), leucine (2.82-fold), lysine (1.35-fold), and cysteine (2.07-fold) was observed over the period of time taken for complete degradation (24 h). The obtained amino acid concentrations are greater than those stated previously (Jeong et al., 2010; Fang et al., 2013; Ramakrishna et al., 2017; Peng et al., 2019; Supplementary Table 1). Tyrosine, leucine, valine, and phenylalanine are essential amino acids that cannot be synthesized in the body. Also, sulfur-containing methionine and cysteine along with tyrosine, phenylalanine, and threonine are important for feather and hair keratin synthesis, whereas amino acid arginine contributes to cats' urea cycle. Hydrolyzed chicken feathers thus have a prodigious potential to produce amino acids, which can be further utilized as animal protein supplements (Peng et al., 2019).

**Table 3:** The concentration of amino acids detected in feather hydrolysate after 12 and 24 h of degradation.

**Amino acid**	**Concentration (μmol/mL)**		
	**0 h**	**12 h**	**24 h**
Aspartic acid	0.29 ± 0.06	0.41 ± 0.05	0.71 ± 0.07
Glutamic acid	2.94 ± 0.17	3.73 ± 0.22	3.13 ± 0.25
Serine	4.48 ± 0.51	5.52 ± 0.81	5.46 ± 0.21
Histidine	0.51 ± 0.05	0.57 ± 0.05	0.62 ± 0.04
Glycine	14.97 ± 0.84	12.75 ± 0.80	12.51 ± 0.97
Threonine	3.62 ± 0.25	3.81 ± 0.19	3.95 ± 0.62
Alanine	16.27 ± 0.27	14.92 ± 0.75	14.06 ± 0.47
Arginine	1.32 ± 0.09	1.48 ± 0.11	1.92 ± 0.10
Tyrosine	5.29 ± 0.21	9.34 ± 0.44	26.87 ± 0.47
Valine	2.16 ± 0.13	7.13 ± 0.16	14.55 ± 0.81
Methionine	12.25 ± 0.87	15.49 ± 0.70	28.45 ± 0.69
Phenylalanine	1.15 ± 0.07	1.61 ± 0.08	3.78 ± 0.37
Isoleucine	3.41 ± 0.14	5.20 ± 0.41	9.56 ± 0.25
Leucine	4.99 ± 0.60	8.35 ± 0.73	14.10 ± 0.41
Lysine	6.88 ± 0.81	7.96 ± 0.18	9.31 ± 0.25
Cysteine	2.41 ± 0.09	3.10 ± 0.21	4.99 ± 0.20
Proline	ND	ND	ND
Tryptophan	ND	ND	ND
Glutamide	ND	ND	ND
Asparagine	ND	ND	ND

### Cytotoxicity of Protein Hydrolysate on Mammalian Cell Lines

Metabolism of nutrients and digestives (amino acids, glucose, and fatty acids) is performed by liver tissue, which encompasses essential enzymes for such requisite functions (Sadi et al., 2015). Also, hepatocellular carcinoma (liver cancer) is a major global concern and therefore HepG2 cells were used for *in vitro* cytotoxicity evaluation of hydrolyzed feathers. Furthermore, cytotoxicity evaluation of an enzyme is extremely important for its commercialization/applicability, and limited attempts are scientifically reported in this regard. Our cytotoxicity data revealed that in *in vitro* conditions 0.09% of protein hydrolysate has very less/negligible cytotoxicity (1.02%), whereas an increase in toxicity was observed at higher concentrations (Table 4). As a hydrolyzed feather at 0.09% concentration exhibited negligible cytotoxic effect as compared to the positive control, it might be explored as an efficient fertilizer and animal feed supplement at low concentrations along with several industrial applications. However, *in vivo* animal modeled examination is essential to assess any allergic or immunomodulatory reaction of the hydrolyzed feather.

**Table 4:** Cytotoxicity evaluation of protein hydrolysate (0.09−50%) using MTT assay in HepG2 cells.

**Compound**	**Concentration**	**Cytotoxicity (%)**
Positive control (doxorubicin hydrochloride)	0.10 μM	49.10 ± 2.19
	1.00 μM	75.62 ± 2.67
	5.00 μM	81.52 ± 3.72
Protein hydrolysate (cell-free supernatant)	0.09%	1.02 ± 0.08
	0.25%	2.51 ± 0.06
	1.00%	4.60 ± 0.14
	1.75%	10.20 ± 0.53
	2.50%	18.29 ± 0.83
	5.00%	21.29 ± 0.99
	10.00%	35.21 ± 1.59
	25.00%	59.21 ± 2.65
	50.00%	61.29 ± 2.36

## Conclusion

Agro-environmental organic waste valorization to beneficial produce is highly essential for sustainable development and has extensively nurtured the bio-economy worldwide. Henceforth, keratinase by *B. pacificus* RSA27 is a novel and efficient eco-friendly alternative for bio-converting chicken feathers into nutritive protein hydrolysate. Scanning electron microscopy has evidently displayed the keratinase potential of chicken feather hydrolysis pattern over time. The protease showed significant keratinolytic activity even at extreme temperature (20–80°C) and pH range (3–12). Here, observations suggest that the keratinase will retain its activity even in the mammalian digestive tract as there is no major loss in its activity even at extreme acidic conditions of pH 3–6. Also, the protease showed significant activity with divalent/monovalent metal ions and surfactants, imperative for optimum production/utility. The keratinase was observed as a serine protease with complete activity inhibition with PMSF (0.09%). *K*_m_ and *V*_max_ values of 5.69 mg/ml and 142.40 μg/ml/min indicate significant enzyme-substrate affinity/biocatalysis. Deduced sequence/structural analysis confirms its novelty with distinct residues, active sites, and high-affinity Ca1-binding site (at diverse positions) when compared with homologous enzymes. Scale-up revealed complete feather hydrolysis with significant activity (789 U/ml) and protein content (153.97 μmol/ml), indicating its substantial use in numerous biotechnological applications. Conclusively, effectual feather degradation by novel keratinase supports the prospective of recalcitrant keratinous waste valorization into valuable produce.

## Data Availability Statement

The datasets presented in this study can be found in online repositories. The names of the repository/repositories and accession number(s) can be found in the article/Supplementary Material.

## Author Contributions

RS contributed to the conception and design of the study. CS conducted the experiments and wrote the manuscript. ST assisted CS in performing experiments and manuscript writing. RS, AO, and JM supervised the study. All authors contributed to manuscript revision, read, and approved the submitted version.

## Funding

The research has been financially supported by the Indian Council of Agricultural Research (ICAR) under F. No. AS/22/5/2018-ASR-IV.

## Conflict of Interest

The authors declare that the research was conducted in the absence of any commercial or financial relationships that could be construed as a potential conflict of interest.

## Publisher's Note

All claims expressed in this article are solely those of the authors and do not necessarily represent those of their affiliated organizations, or those of the publisher, the editors and the reviewers. Any product that may be evaluated in this article, or claim that may be made by its manufacturer, is not guaranteed or endorsed by the publisher.
