# Supplementary material for: Chicken Feather Waste Valorization Into Nutritive Protein Hydrolysate: Role of Novel Thermostable Keratinase From Bacillus pacificus RSA27
Source: Front Microbiol. 2022 Apr 25;13:882902. doi: 10.3389/fmicb.2022.882902 (PMC9083118; doi:10.3389/fmicb.2022.882902)
Supplement: Supplementary file 1 [file Data_Sheet_1.docx]

Supplementary Material

## Supplementary Figures


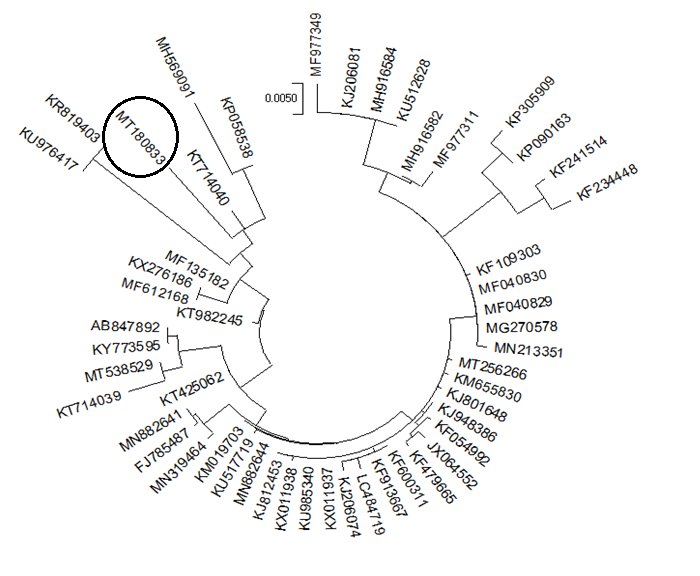


**Supplementary Figure 1.** Phylogenetic tree showing the relation of *Bacillus pacificus* RSA27 (encircled) with some inter-related *Bacillus* species generated using maximum likelihood methodology. The evolutionary distance of 0.0050 is indicated by the bar.

**
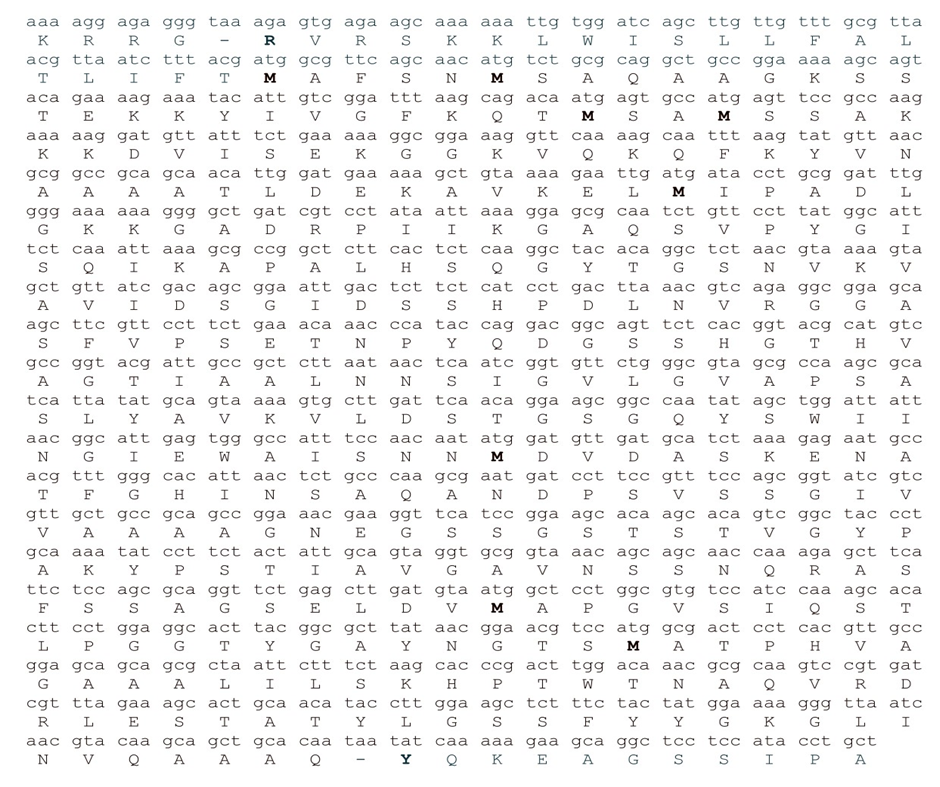
**

**Supplementary Figure 2.** *Ker* gene translation into protein sequence by ExPASY software.


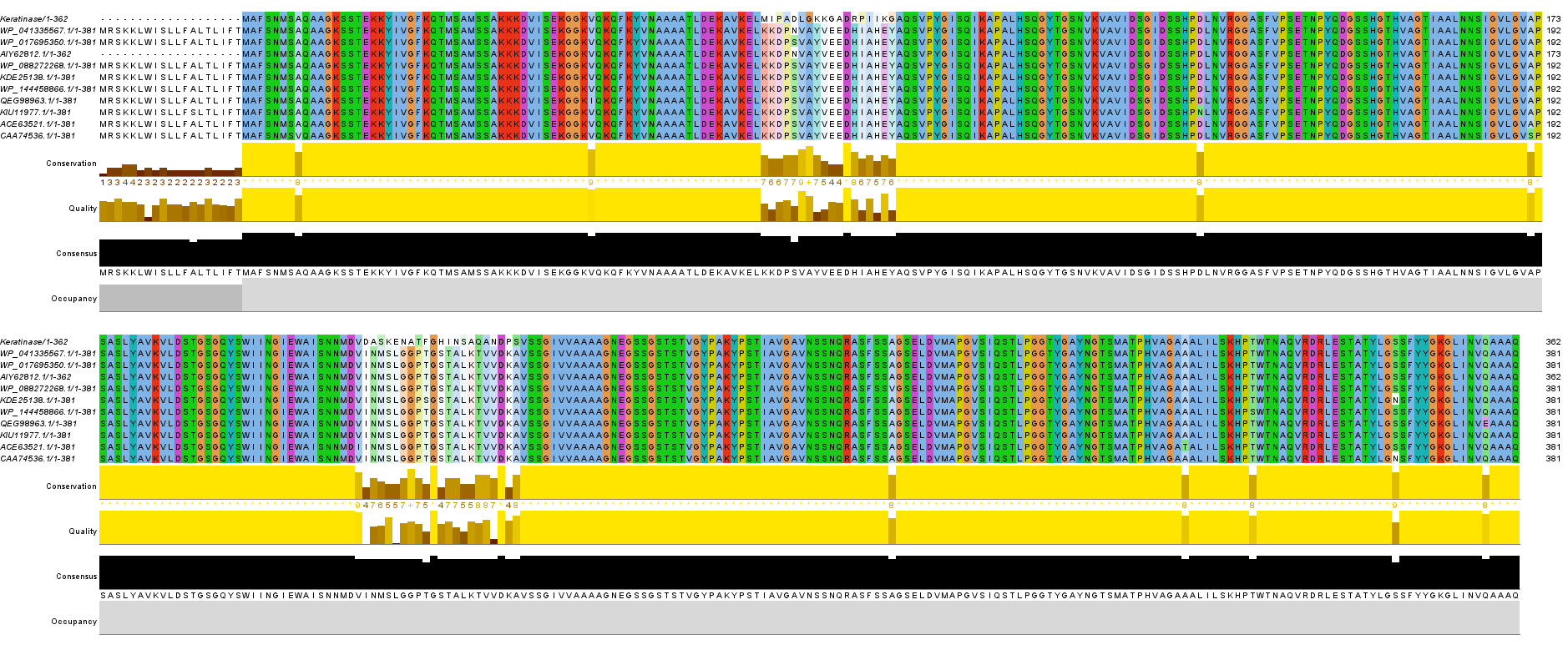


**Supplementary Figure 3.** Multiple sequence alignment of keratinase with top 10 closest homologous enzymes. Keratinase from *Bacillus pacificus* RSA27, Subtilisin AprE from *Bacillus subtilis* (WP_041335567.1), MULTISPECIES: Subtilisin AprE from *Bacillus* (WP_017695350.1), Keratinase from *Bacillus subtilis* (AIY62812.1), Subtilisin AprE from *Bacillus subtilis* (WP_088272268.1), Peptidase S8 from *Bacillus subtilis* (KDE25138.1), Subtilisin AprE from *Bacillus* sp. LM 4-2 (WP_144458866.1), Fibrinolytic enzyme AprE from *Bacillus subtilis* (QEG98963.1), Subtilisin from *Bacillus subtilis* (KIU11977.1), Fibrinolytic enzyme precursor from *Bacillus* sp. ZLW-2 (ACE63521.1) and Subtilisin E precursor from *Bacillus subtilis* subsp. *subtilis* str. 168 (CAA74536.1).

**Supplementary Table 1.** Comparative analysis with reported concentration of amino acids detected in feather hydrolysate.

| **Amino acid** | **Present study (µmoles/ml) (24 h)** | **Peng et al. 2019 (mg/L)**  **(48 h)** | **Ramakrishna et al. 2017 (mg/L)**  **(48 h)** | **Fang et al. 2013 (mg/L)**  **(48 h)** | **Jeong et al. 2010**  **(µM)**  **(96 h)** |
| --- | --- | --- | --- | --- | --- |
| **Aspartic acid** | 0.71 ± 0.07 | 15.55 ± 1.09 | 1.46 | 2.05 | 7.7 |
| **Glutamic acid** | 3.13 ± 0.25 | 20.19 ± 0.13 | 2.23 | 5.57 | 6.7 |
| **Serine** | 5.46 ± 0.21 | 14.61 ± 0.18 | 9.84 | 0.04 | 11.9 |
| **Histidine** | 0.62 ± 0.04 | 0 | 0.14 | 0.15 | 24.5 |
| **Glycine** | 12.51 ± 0.97 | 14.34 ± 0.24 | 7.61 | 4.05 | 8.4 |
| **Threonine** | 3.95 ± 0.62 | 15.74 ± 0.22 | 0 | 0.12 | 24.4 |
| **Alanine** | 14.06 ± 0.47 | 10.05 ± 0.28 | 0.69 | 0.51 | 5 |
| **Arginine** | 1.92 ± 0.10 | 22.13 ± 0.49 | 3.55 | 0.03 | 0 |
| **Tyrosine** | 26.87 ± 0.47 | 171.53 ± 0.42 | 0 | 5.33 | 0 |
| **Valine** | 14.55 ± 0.81 | 207.51 ± 0.29 | 2.31 | 2.24 | 4.7 |
| **Methionine** | 28.45 ± 0.69 | 0.85 ± 0.41 | 7.24 | 0.92 | 31.5 |
| **Phenylalanine** | 3.78 ± 0.37 | 183.73 ± 0.21 | 0 | 14.63 | 0 |
| **Isoleucine** | 9.56 ± 0.25 | 72.96 ± 0.49 | 15 | 0.05 | 0 |
| **Leucine** | 14.10 ± 0.41 | 126.61 ± 0.73 | 0 | 0.02 | 4 |
| **Lysine** | 9.31 ± 0.25 | 20.94 ± 0.79 | 10.81 | 0.28 | 0 |
| **Cysteine** | 4.99 ± 0.20 | 0 | 0 | 2.03 | 15.4 |
